# Latent profile analysis and influencing factors of health literacy among family caregivers of cancer patients

**DOI:** 10.3389/fpubh.2026.1807631

**Published:** 2026-09-15

**Authors:** Fanjiao Meng, Xinyu Wang, Shujuan Chen, Wei Zhou, Yihong Jiang, Jinhong Yang

**Affiliations:** 1 School of Nursing, Shandong Second Medical University, Weifang, Shandong, China; 2 Department of Neurology, Qingdao Women and Children’s Hospital, Qingdao, Shandong, China; 3 Department of Neurology, Weifang People’s Hospital (The First Affiliated Hospital of Shandong Second Medical University), Weifang, Shandong, China; 4 Department of Oncology, Weifang People’s Hospital (The First Affiliated Hospital of Shandong Second Medical University), Weifang, Shandong, China

**Keywords:** cancer, family caregivers, health literacy, latent profile analysis, social support

## Abstract

**Objective:**

To understand the current status, categorical characteristics, and influencing factors of health literacy among family caregivers of cancer patients, providing evidence and guidance for enhancing caregivers’ health literacy and caregiving capabilities to improve cancer patients’ quality of life.

**Methods:**

This study employed convenience sampling to select 287 family caregivers of cancer patients admitted to a tertiary-level Class A hospital in Weifang City between February and August 2025. A cross-sectional survey was conducted using a demographic questionnaire, the Chinese version of the Cancer Caregiver Health Literacy Scale, the General Self-Efficacy Scale, the Caregiver Sense of Efficacy Scale, and the Perceived Social Support Scale. Data analysis employed latent profile analysis of health literacy among family caregivers using Mplus 8.0 software, supplemented by descriptive analysis, univariate analysis, and multivariate logistic regression analysis via SPSS 25.0 to explore factors influencing health literacy.

**Results:**

A total of 271 valid questionnaires were retrieved. The mean health literacy score among family caregivers of cancer patients was 134 (120, 153), indicating a moderate level. The dimension “Processing Health Information” had the lowest mean score of 12 (9, 12). The dimension “Understanding Patient Needs and Preferences” scored highest at 18 (18, 22). Based on latent profile analysis, family caregivers of cancer patients were categorized into three groups: low health literacy (49.8%), moderate health literacy (25.4%), and high health literacy (24.7%). Multivariate logistic regression analysis revealed that age (OR = 9.445, 95% CI: 1.646–54.196), educational attainment (OR = 0.50, 95% CI: 0.003–0.777), occupation (OR = 0.116, 95% CI: 0.021–0.641), and other factors were significantly associated with health literacy categories among family caregivers of cancer patients (*p* < 0.05).

**Conclusion:**

The overall health literacy level among family caregivers of cancer patients was moderate, with nearly half (49.8%) falling into the low health literacy group, though significant heterogeneity existed. Caregivers’ health literacy was influenced by multiple factors, including age, educational attainment, economic status, self-efficacy, social support, and caregiving experience.

## 1 Introduction

Cancer has become one of the leading causes of death worldwide, severely compromising public health and placing a heavy burden on families and societies. According to statistics, approximately 19.976 million new cancer cases and 9.744 million cancer deaths were recorded globally in 2022. Asia accounted for 49.2% of new cancer cases and 56.1% of cancer deaths worldwide (1). By 2025, global new cancer cases are projected to increase by 77% compared to 2022, reaching 28.4 million cases (2). In 2022, China recorded approximately 4.57 million new cancer cases, accounting for about 24.2% of the global total (3). Driven by accelerated population aging, environmental pressures arising from industrialization and urbanization, and the prevalence of unhealthy lifestyles, China’s cancer burden will continue to grow, and the scale of the cancer patient population will further expand (4). As a chronic disease, cancer is characterized by recurrent episodes, prolonged treatment cycles, and high recurrence rates (5). Throughout the protracted battle against cancer, patients endure not only physical suffering but also immense psychological stress (6). Caregivers, serving as patients’ closest supporters, shoulder daily nursing responsibilities while bearing significant pressure regarding medical decisions and disease outcomes (7). Statistics from the World Health Organization (WHO) indicate that approximately 349 million people worldwide provide care for dependent individuals. These patients cannot independently manage their treatment effectively and rely heavily on the support and assistance of others (8). According to the National Alliance for Caregiving (NAC), approximately 3.18 million caregivers support cancer patients in the United States, with most cancer patients relying on caregiver support and care (9). In this study, caregivers refer specifically to unpaid informal family caregivers, primarily including spouses, parents, children and close friends (10–12). Caregivers play a crucial role in the daily care of cancer patients, providing emotional support and assisting the medical team in managing disease progression (13–15). Depending on the patient’s condition, caregivers also undertake a range of medical care tasks, such as wound care, medication management, symptom control, and nutritional support (16–18). Additionally, caregivers play a key role in health decision-making, sharing health information, and facilitating complex medical procedures (19).

Health literacy refers to an individual’s ability to access, understand, and apply health information to promote and maintain health (20, 21). Caregiver health literacy is defined as the ability to access, evaluate, understand, and apply health information and healthcare services to participate in patient medical and care decisions, thereby improving patient health outcomes and quality of life (22). The level of caregiver health literacy plays a decisive role in their ability to effectively fulfill caregiving responsibilities, impacting not only patient health outcomes but also their quality of life and the quality of healthcare services. Research indicates that when caregivers of cancer patients lack sufficient health literacy, they often avoid discussing cancer-related information and fail to effectively convey health messages (23). This leads to increased patient hospitalization rates and heightened economic burdens, ultimately affecting patients’ quality of life and health outcomes (24).

Caregivers’ health literacy is crucial in cancer patient care, yet they face persistent challenges such as difficulty accessing health information, cancer information overload (25), lack of understanding about disease information and healthcare systems, and inadequate communication with patients and medical staff—all of which hinder effective health management (26). Therefore, understanding the current state of health literacy among family caregivers of cancer patients and its influencing factors, while developing effective interventions to enhance caregiver health literacy, holds significant practical importance for improving the quality of care and health outcomes for cancer patients.

## 2 Subjects and methods

### 2.1 Participants

A cross-sectional design was employed in this study. This study selected family caregivers of cancer patients hospitalized at a tertiary-level Class A hospital in Weifang City between February 2025 and August 2025 as research subjects. All enrolled patients spanned clinical stage I to IV, and the detailed distribution of disease stages among participants is summarized in Table 1. This hospital was selected for the following rationales: (1) It is the largest comprehensive oncology center in Weifang, treating all pathological types of malignant tumors and recruiting caregivers from urban, suburban and rural regions across the city, forming a diverse sample pool. (2) The department has sound follow-up systems and completed ethical approval for this questionnaire survey. However, it must be emphasized that single-center convenience sampling inherently restricts external validity, and the sample cannot fully represent family caregivers of cancer patients nationwide.

**Table 1:** General information form for caregivers of cancer patients (*n* = 271).

Variable	Group	Frequency	Percentage (%)
Sex	Male	142	52.4
	Female	129	47.6
Age (years)	≤44	150	55.4
	45 ~ 59	77	28.4
	≥60	44	16.2
Place of residence	City	116	42.8
	Town	59	21.8
	Rural	96	35.4
The relationship with the patient	Spouse	82	30.3
	Children	143	52.8
	Parents	38	14.0
	Siblings	8	3.0
Educational level	≤Primary school	30	11.1
	Junior high school	65	24.0
	Senior high school	68	25.1
	Specialist degree	97	35.8
	Graduate students and above	11	4.1
Marital status	Married	249	91.9
	Unmarried	19	7.0
	Divorced	3	1.1
Occupation	Farmer	48	17.7
	Civil servant	49	18.1
	Others	174	64.2
Monthly per capita income (yuan)	<3,000	44	16.2
	3,000 ~ 5,999	98	36.2
	6,000 ~ 8,999	88	32.5
	≥9,000	41	15.1
Caregiving experience	Yes	106	39.1
	No	165	60.9
Patient care time (years)	≤1	154	56.8
	1 ~ 3	82	30.2
	>3	35	12.9
Self-reported health status	Good	173	64.3
	Generally	96	35.7
Cancer types	Lung	83	30.6
	Colorectal	61	22.5
	Gynecological	25	9.2
	Gastric	23	8.5
	Esophageal	7	2.6
	Liver	24	8.9
	Pancreatic	19	7.0
	Breast	17	6.3
	others	12	4.4
Patient’s medical history	≤6 months	101	37.4
	6 months ~1 year	51	51.0
	>1 year	118	43.7
Disease staging	I	10	3.7
	II	34	12.5
	III	94	34.7
	IV	133	49.1
Know the condition	No	40	14.8
	Part	100	37.0
	Completely	130	48.1

*Inclusion criteria*: (1) Caregivers of patients with histopathologically confirmed cancer, aged ≥18 years, without psychiatric disorders or severe cognitive impairment; (2) involved in medical and nursing decisions and responsible for primary patient care tasks; (3) aged ≥18 years; (4) weekly caregiving time ≥40 h; (5) possessed adequate verbal comprehension and logical reasoning, as briefly assessed by trained researchers via simple face-to-face conversation before questionnaire administration; participants unable to follow basic oral questions were excluded; (6) provided informed consent and voluntarily participated in the study.

*Exclusion criteria*: (1) Caregivers receiving monetary compensation; (2) non-relatives of the patient; (3) current cancer patients or those with a history of cancer; (4) previous participation in similar studies.

### 2.2 Sample size estimation

As this was an exploratory study and no prior effect size estimates were available for this population and measurement tool, we adopted a comprehensive approach to determine sample size. First, following Kendall’s method, the sample size was estimated as 5 to 10 times the number of independent variables (*n* = 21), yielding a range of 126–252 after accounting for 20% attrition (27). Second, for latent profile analysis, simulation studies recommend approximately 50 participants per class for stable estimation (28); with an expected three-class solution, at least 150 participants were required. Third, when BIC is used as the primary index for class selection, a total sample size of at least 200 is recommended (29). Based on these criteria, we recruited 271 participants. To verify the adequacy of our sample size, we conducted a post-hoc power analysis. The research received approval from the hospital ethics committee (Ethics Approval No.: KYLL20240204-4). All participants voluntarily consented to the study and signed informed consent forms.

### 2.3 Survey instruments

#### 2.3.1 General information questionnaire

Developed by the researchers, this questionnaire comprises two sections. ① General information of family caregivers for cancer patients: age, gender, place of residence, relationship to the patient, educational attainment, marital status, occupation, average monthly household income, caregiving experience, duration of caregiving, self-reported health status, etc. ② General information about the cancer patient: cancer type, disease duration, disease stage, awareness of diagnosis, etc.

#### 2.3.2 Health Literacy of Caregiver Scale-Cancer (HLCS-C)

This study employed the Chinese version of the Health Literacy Scale for Caregivers of Cancer Patients, developed and revised by Yuen et al. (28, 29) and adapted into Chinese by Lin et al. (30, 31). The scale encompasses 10 dimensions with a total possible score range of 36–176. Dimensions 1–8 utilize a 4-point Likert scale ranging from “Strongly Disagree” (0 points) to “Strongly Agree” (4 points), Dimensions 9 and 10 employ a 5-point Likert scale ranging from “Unable to complete” to “Very easy,” scored from 0 to 4. Higher scores indicate greater health literacy among caregivers. In this study, Cronbach’s alpha coefficients for each dimension ranged from 0.872 to 0.925, with an overall scale Cronbach’s alpha of 0.969.

#### 2.3.3 General Self-Efficacy Scale (GSES)

This study employed the Chinese version of the General Self-Efficacy Scale developed by Schwarzer et al. (32) and adapted by Wang et al. (33). The scale comprises 10 items scored using a 4-point Likert scale ranging from 1 (“completely untrue”) to 4 (“completely true”), with a total score range of 10 to 40, with higher scores indicating stronger self-efficacy. In this study, the Cronbach’s alpha coefficient for this scale was 0.948.

#### 2.3.4 Perceived Social Support Scale (PSSS)

This study employed the Chinese version of the Perceived Social Support Scale developed by Cohen et al. (34) and translated by Jiang et al. (35). The scale comprises 12 items across 3 dimensions, using a Likert scale ranging from 1 (“Strongly Disagree”) to 7 (“Strongly Agree”), with a total score range of 12 to 84. Higher scores indicate greater perceived social support. In this study, the Cronbach’s *α* coefficient for this scale was 0.953.

#### 2.3.5 Positive Aspects of Caregiving Scale (PAC)

This study employed the Chinese version of the Positive Aspects of Caregiving Scale developed by Tarlow et al. (36) and translated by Zhang et al. (37). This scale comprises two dimensions: Self-Affirmation (5 items) and Life Expectancy (4 items), using a 5-point Likert scale ranging from 1 (“Strongly Disagree”) to 5 (“Strongly Agree”), with a total score range of 9 to 45. Higher scores indicate stronger positive aspects of caregiving. In this study, the Cronbach’s alpha coefficient for this scale was 0.953.

### 2.4 Data collection and control methods

Prior to the survey, two responsible nurses and two graduate students underwent standardized training to ensure they mastered uniform, standardized instructions. This enabled them to clearly explain the purpose, significance, and key points of questionnaire completion to participants. After obtaining verbal permission from hospitalized cancer patients, investigators distributed paper questionnaires to their primary family caregivers after collecting written informed consent signed by caregivers themselves. A small proportion of eligible caregivers declined to participate in the survey, primarily owing to heavy daily care load and limited spare time. Caregivers were encouraged to complete the questionnaire independently, with completed forms collected immediately. For caregivers unable to complete the questionnaire independently due to advanced age, low educational attainment, or visual impairment, researchers assisted through one-on-one questioning. After collection, two graduate students will conduct dual verification. Any omitted, duplicated, or questionable responses will be promptly investigated. Following data entry, 20% of questionnaires will be randomly selected for cross-checking to further ensure data authenticity and reliability.

### 2.5 Statistical methods

Data entry was performed using Excel 2021 with double-entry verification to ensure accuracy. Mplus 8.0 was employed to construct latent profile models of health literacy among cancer patient caregivers. Scores from each dimension of the Chinese version of the Health Literacy Computerized Scale (HLCS-C) served as manifest variables, with 1 to 5 latent profiles sequentially fitted to classify literacy levels. Model fit evaluation employed AIC, BIC, aBIC, entropy, LMR-LRT, and BLRT indices (38): Lower values of AIC, BIC, and aBIC indicate better model fit. Entropy values closer to 1 suggest higher classification accuracy (Entropy > 0.8 indicates 90% classification precision). *p* < 0.05 in LMR-LRT and BLRT tests indicates that the *k*-class model is superior to the *k*-1-class model (39). The optimal model was selected based on practical significance.

Statistical analysis performed using SPSS 25.0: For general data, the Shapiro–Wilk test was used to assess the normality of continuous variables. Normally distributed variables were described as mean ± standard deviation, while non-normally distributed variables were described as median (P25, P75). Categorical variables were described as frequency and percentage. Using latent categories as grouping variables, non-normally distributed continuous and ordinal variables underwent Kruskal-Wallis nonparametric tests, while categorical variables underwent chi-square tests. Discrete-logistic regression was used to analyze factors influencing latent profiles of health literacy. *p* < 0.05 was considered statistically significant. A post-hoc power analysis using G*Power 3.1 demonstrated that, based on the observed model fit (Cox & Snell *R*^2^ = 0.528, *f*^2^ = 1.118) with 21 predictors, *α* = 0.05, and *N* = 271, the achieved power was 0.81, confirming adequate statistical power. Before conducting the multinomial logistic regression analysis, Pearson correlation analysis was performed to examine the correlations among all independent variables. A correlation coefficient threshold of < 0.7 was used to indicate no strong linear relationship between predictors, suggesting no significant multicollinearity.

## 3 Result

A total of 287 questionnaires were distributed to family caregivers of cancer patients meeting the inclusion criteria, with 285 successfully returned. After screening to exclude incomplete responses, those exhibiting repetitive answering patterns, or internal logical inconsistencies, 271 valid questionnaires were ultimately obtained, yielding a valid response rate of 95.08%.

### 3.1 Common method bias test

Given that all data in this study were derived from patient self-reports, there exists a potential risk of common method bias. Therefore, prior to formal data analysis, this study employed unrotated exploratory factor analysis on all scale items using SPSS 25.0 and assessed common method bias in the data via Harman’s single-factor test (40). Results showed that unrotated principal component analysis extracted 11 factors with eigenvalues greater than 1. The first factor explained 37.391% of total variance, below the 40% critical threshold (41), indicating that common method bias had no significant impact on this study.

### 3.2 Sample characteristics

Among the 271 family caregivers of cancer patients, 142 were male and 129 were female. By age, 150 were ≤44 years old, 77 were aged 45–59, and 44 were ≥60 years old. Residence distribution: 116 urban residents, 59 town residents, 96 rural residents; Relationship to patient: 82 spouses, 143 children, 38 parents, 8 siblings; Detailed information is shown in Table 1.

### 3.3 Scores on various scales for family caregivers of cancer patients

#### 3.3.1 Scores on the Health Literacy Scale for family caregivers of cancer patients

The total health literacy score for family caregivers of cancer patients was 134 (120, 153). The total scores and mean scores for each dimension are detailed in Table 2.

**Table 2:** Health Literacy Scale scores among family caregivers of cancer patients [*n* = 271, score, M (P25, P75)].

Dimension	Number of items	Mean score of dimension items	Total dimension score
Determination and initiative in information collection	4	3.25 (3.75, 3.00)	13 (12, 15)
Sufficient information on cancer and cancer management	4	3.00 (2.50, 3.50)	12 (10, 14)
Acquiring relevant information with medical staff support	4	3.00 (3.00, 4.00)	12 (12, 16)
Social support	4	3.25 (3.00, 4.00)	13 (12, 16)
Conducting cancer-related communication with patients	4	3.00 (2.50, 3.50)	12 (10, 14)
Understanding patients’ needs and preferences	6	3.00 (3.00, 3.66)	18 (18, 22)
Self-care	5	3.00 (2.80, 3.40)	15 (14, 17)
Understanding the healthcare system	5	2.80 (2.80, 3.60)	14 (14, 18)
Processing health information	4	3.00 (2.25, 3.00)	12 (9, 12)
Active participation with medical staff	4	3.00 (2.25, 3.00)	12 (12, 15)

#### 3.3.2 Scores on general self-efficacy, perceived social support, and caregiver wellbeing scales among family caregivers of cancer patients

Family caregivers of cancer patients scored 26 (21, 32) on the General Self-Efficacy Scale, 70 (64, 73) on the Perceived Social Support scale, and 36 (32, 43) on the Caregiver WellBeing scale. Scale scores were non-normally distributed and presented as median (*P*_25_, *P*_75_); The value outside parentheses refer to the median, and the two figures inside parentheses represent the 25th and 75th percentiles, respectively.

### 3.4 Results of latent profile analysis of health literacy among family caregivers of cancer patients

This study fitted latent profile models ranging from 1 to 5 categories based on the 10 dimensions of the Health Literacy Scale for Caregivers of Cancer Patients. The model fitting metrics are presented in Table 3. As the number of latent categories increased, the AIC, BIC, and aBIC values progressively decreased. All five models exhibited entropy values exceeding 0.8. When retaining two categories, AIC, BIC, and aBIC values were relatively high. With three categories, the profile information evaluation metrics were comparatively low, and the LMRT reached statistical significance (*p* < 0.05). For four categories, although information evaluation metrics were lower than those for three categories, the LMRT failed to reach statistical significance (*p* > 0.05), and with five categories, the information evaluation index continued to decline while the LMRT similarly failed to reach significance (*p* > 0.05). After comprehensive consideration, the three-category model was selected as the optimal solution, as it had the lowest number of classes with a significant LMRT (*p* = 0.007), whereas the LMRT was non-significant for both the four-class (*p* = 0.225) and five-class (*p* = 0.249) solutions, following established recommendations that prioritize the more conservative LMRT test for class selection.

**Table 3:** Model fit results for the latent profile model of health literacy among family caregivers of cancer patients (*n* = 271).

Category	AIC	BIC	aBIC	Entropy	LMRT (*p*)	BLRT (*p*)	Category probability
1	4759.839	4831.882	4768.468	—	—	—	—
2	3353.669	3465.334	3367.043	0.962	<0.001	<0.001	59.77/40.22
3	3039.752	3191.041	3057.871	0.937	0.007	<0.001	49.81/25.46/24.72
4	2890.119	3081.032	2912.985	0.914	0.225	<0.001	28.41/30.62/19.18/21.77
5	2785.579	3016.115	2813.190	0.910	0.249	<0.001	26.93/28.41/8.85/16.23/19.55

### 3.5 Naming of potential health literacy profiles for family caregivers of cancer patients

The profiles of the three latent categories of health literacy among family caregivers of cancer patients are detailed in Figure 1. Participants were stratified into three subgroups via latent profile analysis implemented in Mplus with all 10 HLCS-C dimension scores as manifest indicators. The mean scores across all health literacy dimension items for caregivers in different profile types exhibit distinct high, medium, and low characteristics. This establishes the hierarchical attributes of the latent health literacy profiles: C1 represents the low health literacy group, C2 the medium health literacy group, and C3 the high health literacy group. C1: 135 cases (49.8%) scored low across all health literacy dimensions, thus named the low health literacy group; C2: 69 cases (25.4%) scored between C1 and C3 across all dimensions, thus named the moderate health literacy group; C3 comprised 67 cases (24.7%), which scored higher than the other two categories across all dimensions and was thus designated as the high health literacy group.

**Figure 1:**
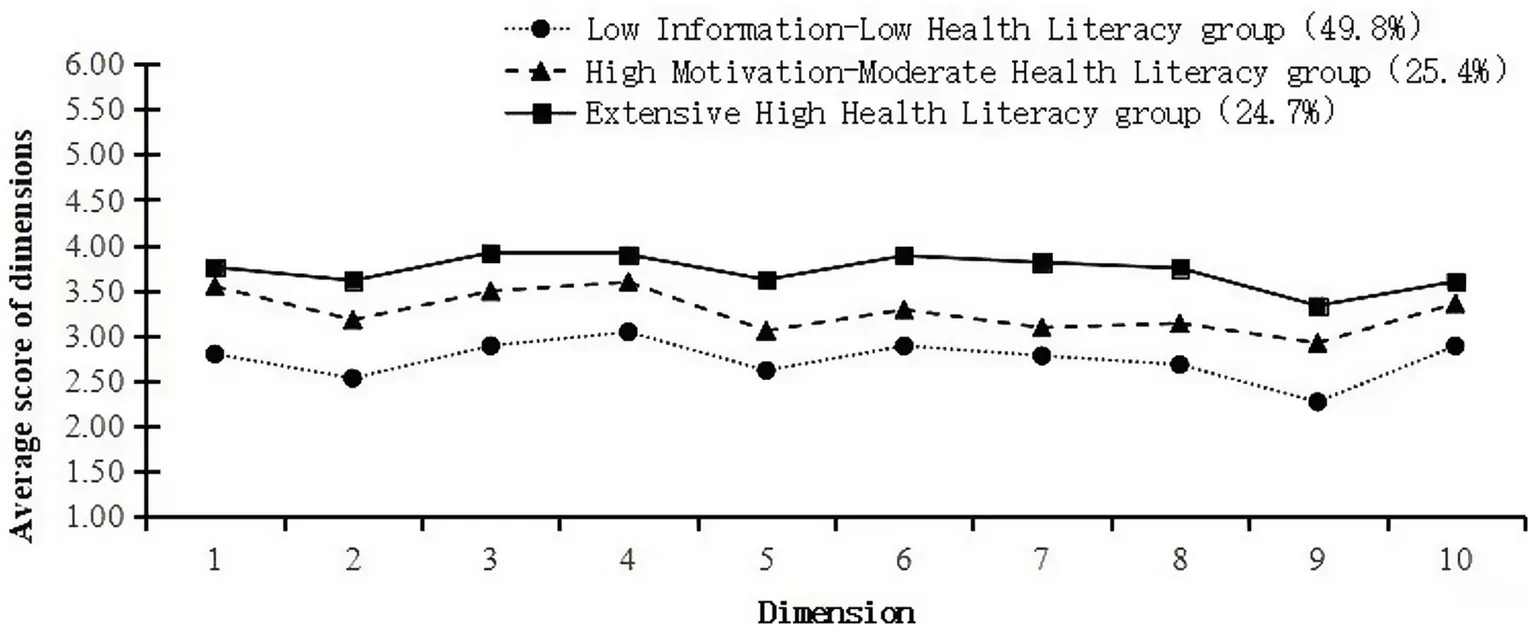
Potential profile characteristics of family caregivers for cancer patients.

### 3.6 Univariate analysis of potential health literacy profiles among family caregivers of cancer patients

Univariate analysis revealed significant differences across the three categories of health literacy among family caregivers of cancer patients for the following variables: age, place of residence, relationship to the patient, educational attainment, occupation, monthly household income per capita, prior caregiving experience, self-rated health status, total general self-efficacy score, total perceived social support score, and total caregiver positivity score (all *p* < 0.05). See Tables 4, 5 for details. Place of residence differed significantly across three profiles (*χ*^2^ = 14.358), (*p* = 0.006). Rural caregivers were overrepresented in the low health literacy group (44.4%), whereas urban participants constituted the majority of medium and high literacy cohorts. Such imbalance was linked to differentiated access to health resources among residents from distinct regions.

**Table 4:** Comparison of general characteristics among family caregivers of cancer patients across different categories [*n* = 271, example (percentage, %)].

Group		C1	C2	C3	Test statistic	*p*
Sex	Male	73 (54.1)	38 (55.1)	31 (46.3)	1.359^*^	*p* = 0.507
	Female	62 (45.9)	31 (44.9)	36 (53.7)		
Age (years)	≤44	54 (40.0)	44 (63.8)	52 (77.6)	35.164^#^	*p*<0.001
	45 ~ 59	43 (31.9)	21 (30.4)	13 (19.4)		
	≥60	38 (28.1)	4 (5.8)	2 (3.0)		
Place of residence	City	44 (32.6)	40 (58.0)	32 (47.8)	14.358^*^	*p* = 0.006
	Town	31 (23.0)	13 (18.8)	15 (22.4)		
	Rural	60 (44.4)	16 (23.2)	20 (29.9)		
The relationship with the patient	Spouse	56 (41.5)	17 (24.6)	9 (13.4)	24.069^*^	*p* = 0.001
	Children	57 (42.2)	43 (62.3)	43 (64.2)		
	Parents	18 (13.3)	6 (8.7)	14 (20.9)		
	Sibling	4 (3.0)	3 (4.3)	1 (1.5)		
Educational level	≤Primary school	27 (20.0)	1 (1.4)	2 (3.0)	39.366^#^	*p*<0.001
	Junior high school	45 (33.3)	14 (20.3)	6 (9.0)		
	Senior high school	27 (20.0)	18 (26.1)	23 (34.3)		
	Specialist degree	34 (25.2)	32 (46.2)	31 (46.3)		
	Graduate students and above	2 (1.5)	4 (5.8)	5 (7.5)		
Marital status	Married	129 (95.6)	63 (91.3)	57 (85.1)	8.939^*^	*p* = 0.063
	Unmarried	4 (3.0)	6 (8.7)	9 (13.4)		
	Married	2 (1.5)	0 (0)	1 (1.5)		
Occupation	Farmer	42 (31.1)	2 (2.9)	4 (11.9)	40.569^*^	*p*<0.001
	Civil servant	16 (11.9)	12 (17.4)	21 (31.3)		
	Others	77 (57.0)	55 (79.7)	42 (62.7)		
Monthly per capita income (yuan)	<3,000	34 (25.2)	4 (5.8)	6 (9.0)		
	3,000 ~ 5,999	56 (41.5)	26 (37.7)	16 (23.9)	20.365^#^	*p*<0.001
	6,000 ~ 8,999	36 (26.7)	24 (34.8)	28 (41.8)		
	≥9,000	9 (6.7)	15 (21.7)	17 (25.4)		
Caregiving experience	Yes	35 (25.9)	30 (43.5)	41 (61.2)	24.127^*^	*p*<0.001
	No	100 (74.1)	39 (45.8)	26 (38.8)		
Patient care time (years)	≤1	72 (53.3)	43 (62.3)	39 (58.2)	1.339^#^	*p* = 0.512
	1 ~ 3	47 (34.8)	19 (20.9)	16 (23.9)		
	>3	16 (11.9)	7 (10.1)	12 (17.9)		
Self-reported health status	Good	68 (51.1)	53 (76.8)	52 (77.6)	19.933^*^	*p*<0.001
	Generally	65 (48.9)	16 (24.6)	15 (22.4)		
Cancer types	Lung	46 (34.1)	22 (31.9)	15 (22.4)	20.830^*^	*p* = 0.228
	Colorectal	32 (23.7)	12 (17.4)	17 (25.4)		
	Gynecological	14 (10.4)	5 (7.2)	6 (9.0)		
	Gastric	8 (5.9)	8 (11.6)	7 (10.4)		
	Esophageal	3 (2.2)	3 (4.3)	1 (1.5)		
	Liver	12 (8.9)	7 (10.1)	5 (7.5)		
	Pancreatic	12 (8.9)	3 (4.3)	4 (6.0)		
	Breast	2 (1.5)	6 (8.7)	9 (13.4)		
	Others	6 (4.4)	3 (4.3)	3 (4.5)		
Patient’s medical history	≤6 months	56 (41.5)	25 (36.2)	20 (30.4)	4.194^#^	*p* = 0.123
	6 months~1 year	23 (17.0)	19 (27.5)	9 (13.6)		
	>1 years	56 (41.5)	25 (36.2)	37 (56.1)		
Disease staging	I	4 (3.0)	2 (2.9)	4 (6.0)	1.510^#^	*p* = 0.470
	II	13 (9.6)	12 (17.6)	8 (11.9)		
	III	48 (35.6)	21 (30.9)	25 (37.3)		
	IV	70 (51.9)	33 (48.5)	30 (44.8)		
Know the condition	No	27 (20.1)	7 (10.1)	6 (9.0)	5.641^#^	*p* = 0.060
	Part	49 (36.6)	29 (42.0)	22 (32.8)		
	Completely	58 (43.3)	33 (47.8)	39 (58.2)		

#Kruskal–Wallis H test; *Chi-square test; C1: Low health literacy group; C2: Moderate Health Literacy Group; C3: High Health Literacy Group.

**Table 5:** Comparison of cancer patients’ family caregivers’ health literacy, general self-efficacy, perceived social support, and caregiver positivity scores across different categories [*n* = 271, Score, M (P25, P75)].

Variable	C1	C2	C3	*H*	*p*
Health literacy total score	120 (114, 128)	143 (138, 148)	165 (158, 168)	223.502	*p*<0.001
General self-efficacy total score	22 (19,26)	29 (24, 33)	34 (29, 40)	95.741	*p*<0.001
Perceived social support total score	68 (59, 70)	72 (65, 77)	76 (72, 83)	70.588	*p*<0.001
Caregiver positive feelings total score	35 (30, 39)	37 (31, 43)	45 (38, 45)	54.180	*p*<0.001

C1: Low health literacy group; C2: Moderate Health Literacy Group; C3: High Health Literacy Group.

### 3.7 Multivariate logistic regression analysis of potential profiles of health literacy among family caregivers of cancer patients

Using the latent categories of health literacy among family caregivers of cancer patients as the dependent variable, multivariate logistic regression analysis was conducted with statistically significant indicators from the univariate analysis as independent variables. The parallelism test yielded *p* < 0.05, failing to meet parallelism criteria. Therefore, a disordinal multinomial logistic regression model was selected. Model fit information indicated a chi-square value of 203.439 (*p* < 0.001), Cox & Snell *R*^2^ of 0.528, and Nagelkerke *R*^2^ of 0.603, confirming good model fit (42). Multivariate analysis revealed that age, place of residence, educational attainment, occupation, per capita monthly household income, prior caregiving experience, general self-efficacy, perceived social support, and caregiver positivity were factors influencing the health literacy category of family caregivers for cancer patients. Compared with the C1 low health literacy group (reference), caregivers aged ≤44 years (OR = 9.445, *p* = 0.012) and 45–59 years (OR = 5.285, *p* = 0.034), those with higher self-efficacy (OR = 1.144, *p* < 0.001), and those with higher perceived social support (OR = 1.061, *p* = 0.012) were more likely to be in the C2 moderate health literacy group; whereas those with primary education (OR = 0.50, *p* = 0.032), a monthly household income between ¥3,000 and ¥5,999 (OR = 0.308, *p* = 0.049), and those engaged in agriculture (OR = 0.116, *p* = 0.014) were less likely to be in the C2 group. Similarly, compared with the C1 group (reference), caregivers aged ≤44 years (OR = 58.100, *p* = 0.001) and 45–59 years (OR = 13.619, *p* = 0.015), those with caregiving experience (OR = 3.786, *p* = 0.004), those with higher self-efficacy (OR = 1.221, *p* < 0.001), and those with higher caregiver positivity (OR = 1.131, *p* = 0.009) were more likely to be in the C3 high health literacy group; whereas those with junior high school education (OR = 0.102, *p* = 0.046), a monthly household income between ¥3,000 and ¥5,999 (OR = 0.225, *p* = 0.036), and those residing in towns (OR = 0.231, *p* = 0.034) were less likely to be in the C3 group. Variable values are detailed in Table 6, and specific results are presented in Table 7.

**Table 6:** Multivariate logistic regression analysis of variable assignments for potential health literacy profiles among family caregivers of cancer patients.

Variable	Assignment
Age (years)	≤44 = 1, 45 ~ 59 = 2, ≥ 60 = 3
Place of residence	city = 1, town = 2, rural = 3
The relationship with the patient	spouse = 1, children = 2 parents = 3 siblings = 4
Educational level	≤Primary school = 1, Junior high school = 2, Senior high school = 3, Specialist degree = 4, Graduate students and above = 5
Occupation	Farmer = 1, Civil servant = 2, others = 3
Monthly per capita income (yuan)	<3,000 = 1,3,000 ~ 5,999 = 2,6,000 ~ 8,999 = 3, ≥ 9,000 = 4
Caregiving experience	Yes = 0, No = 1
Self-reported health status	Goods = 0, Generally = 1
General self-efficacy total score	Substitute the original value
Perceived social support total score	Substitute the original value
Caregiver positive feelings total score	Substitute the original value

**Table 7:** Multivariate logistic regression of factors influencing potential categories of health literacy among family caregivers of cancer patients.

Variable	C1^*^ vs. C2				C1^*^ vs. C3				C2 vs. C3^*^			
	*β*	*p*	OR	95%CI	*β*	*p*	OR	95%CI	*β*	*p*	OR	95%CI
Age (years)												
≤44	2.245	0.012	9.445	1.646 ~ 54.196	4.062	0.001	58.100	5.405 ~ 624.589	−1.817	0.124	0.163	0.016 ~ 1.644
45 ~ 59	1.665	0.034	5.285	1.139 ~ 24.354	2.311	0.015	13.619	1.649 ~ 112.499	−0.947	0.389	0.388	0.045 ~ 3.342
≥60	—	—	—	—	—	—	—	—	—	—	—	—
Place of residence												
City	0.156	0.744	1.169	0.458 ~ 2.984	−0.349	0.536	0.705	0.233 ~ 2.133	0.506	0.325	1.658	0.605 ~ 4.541
Town	−0.898	0.119	0.407	0.131 ~ 1.261	−1.465	0.034	0.231	0.060 ~ 0.892	0.566	0.374	1.762	0.506 ~ 6.142
Rural	—	—	—	—	—	—	—	—	—	—	—	—
The relationship with the patient												
Spouse	0.045	0.969	1.046	0.109 ~ 10.040	0.458	0.757	1.581	0.087 ~ 28.785	−0.413	0.762	0.662	0.046 ~ 9.558
Children	−0.693	0.520	0.500	0.061 ~ 4.122	−0.623	0.647	0.536	0.037 ~ 7.726	−0.069	0.956	0.933	0.078 ~ 11.115
Parents	−1.857	0.114	0.156	0.016 ~ 1.563	−0.986	0.491	0.373	0.023 ~ 6.186	−0.871	0.519	0.418	0.030 ~ 5.916
Siblings	—	—	—	—	—	—	—	—	—	—	—	—
Educational level												
≤Primary school	−2.993	0.032	0.50	0.003 ~ 0.777	−1.940	0.136	0.144	0.011 ~ 1.839	−1.053	0.478	0.349	0.019 ~ 6.407
Junior high school	−1.222	0.221	0.295	0.042 ~ 2.087	−2.282	0.046	0.102	0.011 ~ 0.962	1.060	0.257	2.886	0.463 ~ 18.003
Senior high school	−0.461	0.645	0.631	0.089 ~ 4.471	−0.685	0.530	0.504	0.059 ~ 4.290	0.225	0.792	1.252	0.237 ~ 6.616
Specialist degree	−0.340	0.727	0.712	0.105 ~ 4.809	−0.674	0.527	0.510	0.063 ~ 4.107	0.334	0.680	1.397	0.285 ~ 6.839
Graduate students and above	—	—	—	—	—	—	—	—	—	—	—	—
Occupation				~				~				~
Farmer	−2.153	0.014	0.116	0.021 ~ 0.641	−1.311	0.141	0.269	0.047 ~ 1.546	−0.842	0.430	0.431	0.053 ~ 3.483
Civil servant	−0.317	0.540	0.728	0.264 ~ 2.008	0.545	0.339	1.725	0.564 ~ 5.273	−0.863	0.088	0.422	0.157 ~ 1.137
Others	—	—	—	—	—	—	—	—	—	—	—	—
Monthly per capita income (yuan)												
<3,000	−1.244	0.139	0.228	0.056 ~ 1.496	−0.475	0.611	0.622	0.100 ~ 3.878	−0.770	0.402	0.463	0.077 ~ 2.798
3,000 ~ 5,999	−1.176	0.049	0.308	0.096 ~ 0.995	−1.491	0.036	0.225	0.560 ~ 0.907	0.315	0.589	1.370	0.438 ~ 4.285
6,000 ~ 8,999	−1.078	0.605	0.340	0.104 ~ 1.115	−0.753	0.264	0.471	0.126 ~ 1.766	−0.325	0.548	0.723	0.250 ~ 2.086
≥9,000	—	—	—	—	—	—	—	—	—	—	—	—
Caregiving experience												
Yes	0.521	0.183	1.684	0.782 ~ 3.626	1.331	0.004	3.786	1.532 ~ 9.357	−0.810	0.048	0.445	0.197 ~ 1.007
No	—	—	—	—	—	—	—	—	—	—	—	—
Self-reported health status												
Good	0.604	0.108	1.829	0.877 ~ 3.817	0.335	0.459	1.398	0.576 ~ 3.395	0.269	0.564	1.308	0.525 ~ 3.259
Generally	—	—	—	—	—	—	—	—	—	—	—	—
General self-efficacy total score	0.135	<0.001	1.144	1.069 ~ 1.225	0.200	<0.001	1.221	1.122 ~ 1.329	−0.065	0.092	0.937	0.869 ~ 1.011
Perceived social support total score	0.059	0.012	1.061	1.013 ~ 1.111	0.028	0.349	1.029	0.970 ~ 1.0	0.031	0.295	1.031	0.973 ~ 1.093
Caregiver positive feelings total score	−0.021	0.489	0.979	0.922 ~ 1.040	0.123	0.009	1.131	1.031 ~ 1.240	−0.144	0.002	0.866	0.790 ~ 0.948

C1: Low health literacy group; C2: Moderate Health Literacy Group; C3: High Health Literacy Group; *Reference group; C1 vs. C2, with C1 as the reference; C1 vs. C3, with C1 as the reference; C2 vs. C3, with C3 as the reference.

## 4 Discussion

### 4.1 Analysis of current health literacy status among family caregivers of cancer patients

The study findings indicate that family caregivers of cancer patients achieved an overall health literacy score of 134 (range: 120–153). The dimension with the lowest score was “Processing Health Information,” while the highest score was in the dimension of “Understanding Patient Needs and Preferences.” This finding is consistent with the results of Lin et al. (30) and Wittenberg et al. (43), indicating that family caregivers demonstrate a relatively strong ability to comprehend and respond to the complex health needs of cancer patients. However, significant deficiencies persist in their capacity to access, interpret, and apply health information. This may stem from family caregivers typically being relatives with long-standing, intimate relationships and frequent daily interactions with patients, enabling them to promptly identify and address needs through observation and communication. Due to deep emotional bonds, caregivers are more willing to invest time and effort in understanding patients’ needs and preferences to provide optimal support and care. However, cancer-related medical information is complex, involving multidisciplinary knowledge, and many caregivers lack medical backgrounds, making comprehension difficult. Furthermore, limited access to information sources means caregivers rely on verbal explanations from doctors or basic written materials, lacking systematic, easily understandable health education resources. Therefore, to address the low scores in the “processing health information” dimension, healthcare professionals should provide personalized health education. This includes designing tiered, clear, and easy-to-understand health education resources and utilizing multimedia formats to help caregivers comprehend medical knowledge. Furthermore, information access channels should be expanded by offering multiple avenues such as online resources, health lectures, and detailed written materials to ensure information is both accessible and comprehensible.

### 4.2 Latent profile characteristics of health literacy among family caregivers of cancer patients

This study identified three latent profiles of health literacy among family caregivers of cancer patients through latent profile analysis: low health literacy group, moderate health literacy group, and high health literacy group. This indicates significant individual variation in health literacy among these caregivers. Caregivers in the low health literacy group accounted for 49.8%. This group scored lower in the dimensions of “sufficient cancer and cancer management information” and “processing health information,” with the lowest scores observed in the “processing health information” dimension. This indicates that such caregivers typically lack effective information access channels and exhibit significant deficiencies in obtaining, understanding, and processing cancer-related information. This may be influenced by multiple factors including age, educational attainment, place of residence, household income, caregiving experience, occupation, and social support. Healthcare professionals should pay closer attention to this group of caregivers, strengthen their health education, provide concise and easy-to-understand health education materials, utilize multimedia formats to aid in understanding cancer knowledge, enhance their health literacy and information processing capabilities, and simultaneously establish online health information platforms and hotline consultation services to facilitate caregivers’ access to professional medical advice (44).

Caregivers in the moderate health literacy group accounted for 25.4%. This group scored higher in the dimensions of “determination to gather information,” “proactiveness,” and “active participation alongside healthcare providers,” but remained at an overall moderate level. This indicates that while these caregivers actively engage in information gathering and interactions with healthcare providers, there is still room for improvement in certain health literacy dimensions. For this group, healthcare providers should deliver targeted health education addressing their weaker dimensions, such as “communicating with patients about cancer-related matters” and “processing health information.” Strengthening their training in effective communication and interaction skills with patients will help elevate their overall health literacy, thereby improving care quality and enhancing patient satisfaction and treatment outcomes.

The high health literacy group accounted for 24.7%, indicating that caregivers in this category generally possess higher health literacy. This may be closely related to their higher levels of education, self-efficacy, social support, and positive caregiver identity. Healthcare providers should leverage their role as role models for these caregivers, encouraging high health literacy caregivers to establish supportive and collaborative relationships with low health literacy caregivers. This approach enables them to jointly address caregiving challenges and issues, facilitate the exchange of experiences and emotional support, and ultimately enhance overall care quality. Notably, while the low health literacy group had uniformly lower scores across all dimensions, the most pronounced deficit was concentrated in the “processing health information” dimension, whereas “understanding patient needs and preferences” remained relatively preserved. This dimensional heterogeneity—which cannot be captured by total scores alone—has direct implications for intervention design: it suggests that improving caregivers’ ability to process and apply health information should be the priority target for the low health literacy group, rather than addressing all dimensions uniformly.

### 4.3 Analysis of factors influencing potential categories of health literacy among family caregivers of cancer patients

#### 4.3.1 Age, educational attainment, place of residence, monthly *per capita* household income, occupation

The results of the multivariate logistic regression analysis in this study indicate that compared with caregivers in the low health literacy group, those aged ≤44 and 45–59 years were more likely to be classified into the high and moderate health literacy groups. Compared with older‑adult caregivers, middle-aged and young caregivers demonstrated higher levels of health literacy (30, 45). This phenomenon can be largely explained by differences in digital access and social resources across age cohorts. Older‑adult caregivers, however, may face difficulties in using modern technology, limiting their avenues for acquiring health information. Middle-aged and young caregivers often possess broader social support networks, including colleagues, friends, and community resources, which facilitate their access to and sharing of health information. In contrast, older‑adult caregivers may have more limited social support networks, impacting their health literacy. Additionally, due to work and family responsibilities, middle-aged and young caregivers are generally more attentive to health management and actively engage in health maintenance and oversight. Older‑adult caregivers, however, may exhibit lower motivation for health management due to physical limitations and insufficient health awareness. Healthcare professionals should provide older‑adult caregivers with training on using the internet and smart devices to help them learn how to access and utilize online health resources (46). Design health information platforms and applications that are user-friendly for older adults, ensuring ease of operation and comprehension to enhance their ability to access health information.

Compared to caregivers in the high and medium health literacy groups, those with primary or junior high school education levels are more likely to belong to the low health literacy group. Higher educational attainment among caregivers of cancer patients correlates with higher health literacy levels (47, 48). This may be because caregivers with lower educational attainment often have limited access to information, making it difficult for them to comprehend and utilize complex health information, which results in lower health literacy. Additionally, caregivers with lower educational attainment face restricted access to resources and lack channels to obtain high-quality health resources, further limiting their health literacy. Therefore, healthcare providers should simplify health information for caregivers with lower educational attainment, providing easily understandable health information and avoiding the use of complex medical terminology (49). Simultaneously, health education should be strengthened through multiple channels such as health lectures, informational brochures, and WeChat public account posts to enhance caregivers’ health knowledge and skills.

Compared with the high health literacy group, caregivers residing in townships were more likely to belong to the low health literacy group than patients residing in urban districts (47). This three-category classification of residential location conforms to China’s official administrative division criteria, which can better capture disparities in medical resource availability, health education coverage and caregiving pressure across different living environments than the conventional binary urban–rural grouping adopted in most prior studies. Chi-square test results presented in Table 4 confirmed statistically significant differences in the distribution of caregivers with varying health literacy profiles across city, town and rural residence groups (*p* = 0.006). Compared to caregivers residing in towns, those in urban districts exhibit higher health literacy levels. This may stem from urban caregivers typically having more accessible and extensive information resources, along with greater access to advanced medical facilities and specialized healthcare professionals. These resources provide caregivers with more comprehensive health services and education, facilitating easier access to the latest health information and knowledge. Conversely, townships may offer less accessible information resources, fewer health information dissemination channels, relatively limited medical resources and services, insufficient medical facilities and specialized healthcare personnel, and lower quality and coverage of health services. These factors increase the difficulty for caregivers to obtain health information, thereby impacting their health literacy (50). This suggests healthcare professionals should intensify health education promotion in towns and rural areas. This can be achieved by regularly organizing health lectures, free clinics, and awareness campaigns, while providing illustrated health education materials. Concurrently, mobile healthcare services should be promoted, and the facilities and professional standards of primary healthcare institutions should be enhanced. These measures will ensure the accessibility of high-quality medical services and elevate the health literacy of caregivers in towns and rural areas.

Compared with caregivers in the high and medium health literacy groups, those with a household per capita monthly income of ¥3,000–5,999 were more likely to belong to the low health literacy group than those with an income of ¥9,000 or higher. Household monthly per capita income was divided into four grades (<3,000, 3,000–5,999, 6,000–8,999, ≥9,000 yuan). This four-level grouping standard was formulated based on the local residents’ income distribution and medical expenditure characteristics in Shandong. The three cut-off points of 3,000, 6,000 and 9,000 yuan were set to objectively distinguish gaps in families’ affordability of medical services and accessibility to health information. Caregivers with higher household per capita income demonstrated higher health literacy levels than those with lower household per capita income. Caregivers from low-income households may lack sufficient energy and time to focus on health issues due to limited financial resources, high life pressures, and time constraints, making it difficult for them to access high-quality health services and medical resources (51). Conversely, high-income families may have greater capacity and willingness to participate in health promotion activities and purchase health products and services, which can enhance caregivers’ health literacy (52). This suggests that healthcare professionals can assist caregivers from lower-income households by informing them about available medical insurance policies and medical subsidies to alleviate financial burdens. Simultaneously, developing health support groups or volunteer services can provide caregivers with emotional support and practical advice, ensuring they can effectively access and utilize health information and services.

It should be noted that male caregivers accounted for a slightly higher proportion in this sample. The difference can be attributed to the gendered division of domestic labor in Chinese families. Male relatives are usually responsible for physically strenuous tasks such as patient transfer, inspection accompaniment and hospital formalities, whereas female family members undertake routine housework and childcare. Such differentiated family responsibilities collectively lead to the observed gender distribution of primary caregivers in this study.

Compared to the moderate health literacy group, caregivers whose occupation is farming are more likely to be classified in the low health literacy group (53). Farming caregivers typically have lower educational attainment and may face challenges related to cultural literacy—that is, the ability to access, comprehend, and apply knowledge embedded within the mainstream health system, including an understanding of how medical services are organized, how to navigate healthcare institutions, and how to interpret health-related information from standard sources. For farming caregivers, limited educational background, geographic isolation from medical facilities, and reduced exposure to modern health information channels may jointly contribute to a relative deficiency in this type of cultural knowledge, thereby hindering their effective use of health information and services. Furthermore, constrained economic conditions reduce their capacity to participate in health promotion activities and purchase health services. These factors collectively impact their health literacy levels. This suggests that healthcare providers should minimize the use of excessive medical jargon when communicating with farmer caregivers. When designing health education materials, simpler, more accessible language should be employed, supplemented with multimedia formats such as charts, videos, and audio recordings. This approach makes information more intuitive and memorable, helping caregivers better understand relevant health knowledge and enhance their health literacy levels (49).

#### 4.3.2 Caregiving experience

Compared to those with low or moderate health literacy, caregivers with prior caregiving experience are more likely to belong to the high health literacy group. Caregivers with such experience typically accumulate extensive health-related knowledge and expertise while caring for others, thereby enhancing their health literacy to some extent (54). Healthcare professionals should establish supportive and interactive community or online platforms to encourage caregivers with caregiving experience to share their insights and knowledge. This fosters mutual support among caregivers, enabling those with less experience to learn from others’ experiences and access health information.

#### 4.3.3 General self-efficacy, perceived social support, and caregiver positivity

This study found that general self-efficacy, perceived social support, and caregiver positivity serve as protective factors for health literacy among family caregivers of cancer patients. Compared to the low health literacy group, caregivers in the moderate and high health literacy groups demonstrated higher self-efficacy. Caregivers with high self-efficacy exhibit positive health attitudes, a sense of responsibility, and effective problem-solving abilities. These traits make them more receptive to health knowledge and advice, thereby enhancing their health literacy levels (55, 56). Compared to the low health literacy group, caregivers in the moderate health literacy group demonstrated higher levels of perceived social support. Caregivers with higher levels of perceived social support are able to proactively establish and utilize social support networks, including family, friends, and community resources. They obtain health information, emotional support, and practical advice from these networks, thereby enhancing their health literacy through active social participation and information exchange (30).

Compared to the low health literacy group, caregivers in the moderate health literacy group and the broad high health literacy group exhibit higher levels of caregiver positivity (57). Greater caregiver positivity is linked to more optimistic mindsets and sound psychological status, allowing caregivers to learn and apply health information more efficiently. This suggests healthcare providers should promptly offer caregivers positive feedback and recognition, acknowledging their achievements and efforts in caregiving. Additionally, providers should encourage patients to express gratitude and affirmation toward caregivers, thereby enhancing their self-efficacy and positive feelings, strengthening their confidence in overcoming challenges, and fostering a proactive approach to acquiring health knowledge. Furthermore, helping caregivers establish robust social support networks—including effective utilization of family, friends, and community resources—enables better access to health information and emotional support, thereby improving health literacy levels.

#### 4.3.4 Cultural characteristics of Chinese family caregiving and comparison with international studies

Traditional Chinese cultural norms shape caregivers’ health literacy and distinguish our findings from Western research. First, Confucian filial piety leads adult children to undertake primary unpaid cancer care without systematic professional training, unlike Western institutional professional care systems (58). Second, collectivism and the custom of concealing severe cancer diagnoses create information barriers that reduce caregivers’ motivation to seek disease-related knowledge. Third, hierarchical doctor–patient communication norms discourage less educated and rural caregivers from proactively consulting medical staff for detailed health guidance.

Several key discrepancies exist between this study and overseas work. Western samples are dominated by spousal caregivers with binary urban–rural residence grouping (59), while our participants are mostly adult children, classified by China’s three-tier administrative residential categories (city/town/rural). Foreign studies identify formal community care support as a core protective factor (60), yet our results highlight family emotional support and self-efficacy, due to incomplete grassroots palliative care coverage in China. We adopted five educational strata (primary school and below, junior high, senior high, college, undergraduate and above) matching local population distribution, rather than the simple binary grouping common in Western literature. Such divergences stem from China’s unique cultural values, primary healthcare layout and demographic characteristics.

## 5 Limitations

The participants in this study were exclusively from a hospital in Weifang, China, resulting in a sample with limited representativeness. Future research should address this issue by expanding the sample size and conducting multicenter studies. Finally, the study data were obtained through self-administered questionnaires completed by patient caregivers, which may introduce reporting bias and social desirability bias. Additionally, a formal *a priori* power analysis was not conducted due to the exploratory nature of this study, although the post-hoc power analysis confirmed adequate statistical power for the observed associations. Furthermore, the multinomial logistic regression model contained several subgroup comparisons with small cell sizes, which may have led to inflated odds ratio estimates; these findings should therefore be interpreted with caution. Nevertheless, future studies with larger samples and prospective power calculations are warranted to confirm our findings.

## 6 Conclusion

The findings of this study indicate that the health literacy of cancer patient caregivers is generally at a moderate level, with significant heterogeneity. Nearly half (49.8%) fall into the low health literacy group, with the lowest scores observed in the “processing health information” dimension. Age, educational attainment, place of residence, monthly household income per capita, occupation, prior caregiving experience, general self-efficacy, perceived social support, and caregiver positivity were significantly associated with health literacy categories. However, given the cross-sectional design, these associations are correlational and require further validation.

## Data Availability

The original contributions presented in the study are included in the article/supplementary material, further inquiries can be directed to the corresponding author.
